# Supplementary material for: Proteoglycan SPOCK1 as a Poor Prognostic Marker Promotes Malignant Progression of Clear Cell Renal Cell Carcinoma via Triggering the Snail/Slug-MMP-2 Axis-Mediated Epithelial-to-Mesenchymal Transition
Source: Cells. 2023 Jan 17;12(3):352. doi: 10.3390/cells12030352 (PMC9913795; doi:10.3390/cells12030352)
Supplement: Supplementary file 1 [file cells-12-00352-s001.zip › cells-2128495-supplementary.pdf]

Supplemental Information

Proteoglycan SPOCK1 as a Poor Prognostic Marker Promotes Malignant Progression of Clear Cell Renal Cell Carcinoma via Triggering the Snail/Slug-MMP-2 Axis-Mediated Epithelial-to-Mesenchymal Transition

Yung-Wei Lin, Yu-Ching Wen, Chi-Hao Hsiao, Feng-Ru Lai, Shun-Fa Yang, Yi-Chieh Yang, Kuo-Hao Ho, Feng-Koo Hsieh, Michael Hsiao, Wei-Jiunn Lee * and Ming-Hsien Chien *

* Correspondence: lwj5905@gmail.com (W.-J.L.); mhchien1976@gmail.com (M.-H.C.)

Table S1. Relationship between SPOCK1 mRNA levels and clinicopathological features in 285 patients with papillary renal cell carcinoma (pRCC)

**Characteristic**	**SPOCK1 expression**		***p* value**
	**Low (*n* = 142)**	**High (*n* = 143)**	
Age, years			
< 61	69	66	0.68
≥ 61	73	77	
Gender			
Female	35	40	0.524
Male	107	103	
Stage			
I + II	97	94	0.593
III+IV	31	35	
Tumor T status			
T1+T2	115	109	0.327
T3+T4	27	34	
Lymph node metastasis			
N0	29	21	0.088
N1	9	19	
Unknown	104	103	
Distant metastasis			
M0	51	42	0.151
M1	2	7	
Unknown	89	94	

Figure Legends

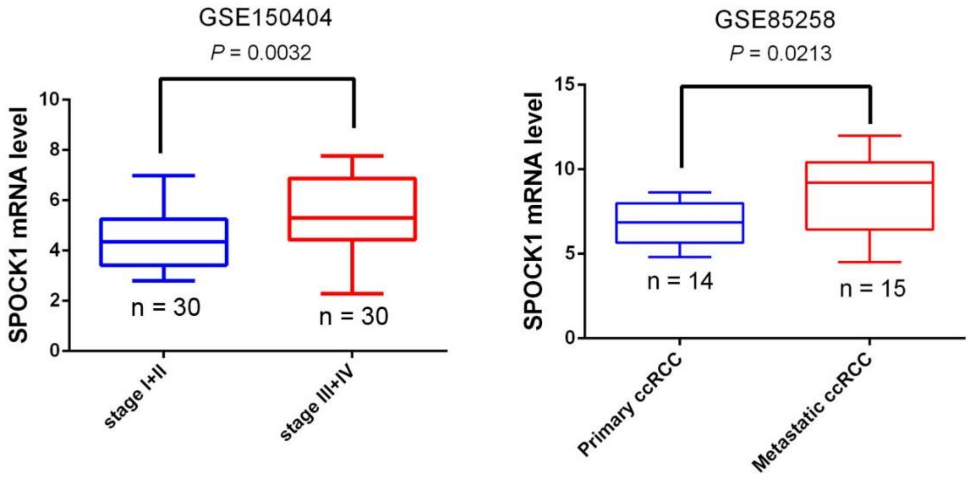

Figure S1. SPOCK1 expression correlates with advanced clinical stages and metastasis in patients with clear cell renal cell carcinoma (ccRCC). *SPOCK1* gene expression levels in ccRCC samples from the GEO were compared according to clinical stage (GSE150404) and distal metastasis (GSE85258). The GSE85258 cohort contained pairs of primary ccRCC tumors and patient-matched pulmonary metastases.

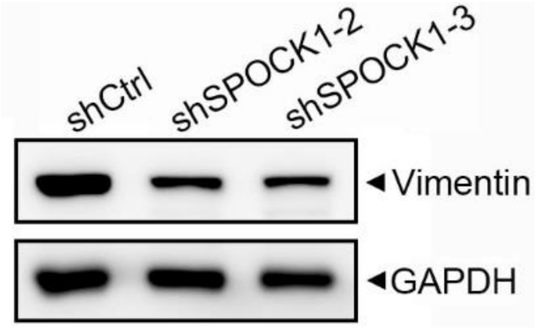

Figure S2. Effects of SPOCK1-knockdown on vimentin expression in clear cell renal cell carcinoma (ccRCC) cells. Caki-1 ccRCC cells were infected with a lentivirus carrying either two specific SPOCK1 shRNAs or shCtrl and subjected to a Western blot analysis to determine vimentin expression. Quantitative results of vimentin proteins were adjusted to GAPDH protein levels.

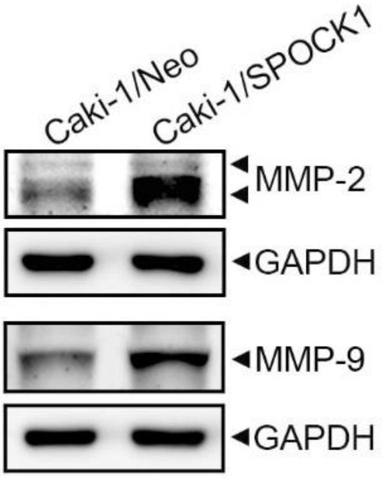

Figure S3. Effects of SPOCK1-knockdown on matrix metalloproteinase (MMP)-2 and MMP-9 expressions in clear cell renal cell carcinoma (ccRCC) cells. Caki-1 ccRCC cells were infected with a lentivirus carrying either a SPOCK1-expressing vector or control vector and were subjected to a Western blot analysis to determine expressions of MMP-2 and MMP-9. Quantitative results of indicated MMP proteins were adjusted to GAPDH protein levels.

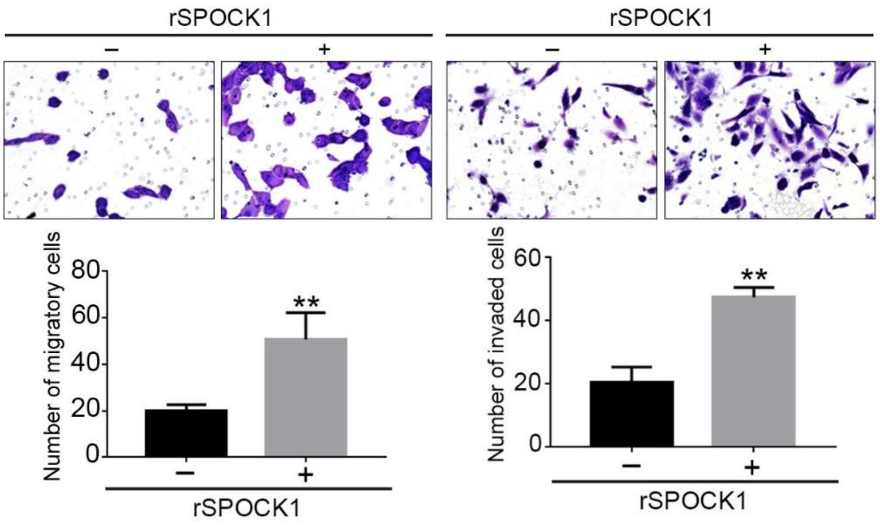

Figure S4. Effect of recombinant (r)SPOCK1 on the migratory and invasive abilities of clear cell renal cell carcinoma (ccRCC) cells. Treatment of Caki-1 cells with the rSPOCK1 protein (1 ng/ml) or vehicle control for 24 h, and the migratory and invasive abilities of cells were respectively determined by transwell migration and Matrigel invasion assays. Data are presented as the mean ± SD of three independent experiments. ** $p < 0.001$, compared to control cells.

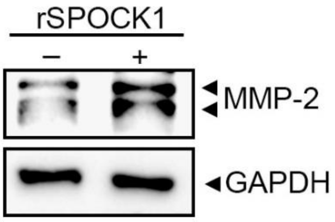

Figure S5. Effects of recombinant (r)SPOCK1 on matrix metalloproteinase (MMP)-2 expression in clear cell renal cell carcinoma (ccRCC) cells. Treatment of 786-O cells with the rSPOCK1 protein (1 ng/ml) or vehicle control for 24 h, and MMP-2 expression levels were detected by Western blotting. Quantitative results of MMP-2 were adjusted to GAPDH levels.

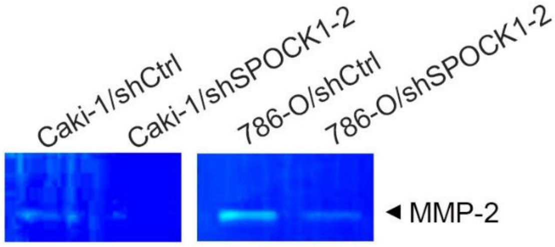

Figure S6. Effects of SPOCK1-knockdown on matrix metalloproteinase (MMP)-2 activity in clear cell renal cell carcinoma (ccRCC) cells. Caki-1 and 786-O cells were infected with a lentivirus carrying either SPOCK1 shRNA or shCtrl and subjected to a gelatin zymography assay to determine MMP-2 activity.

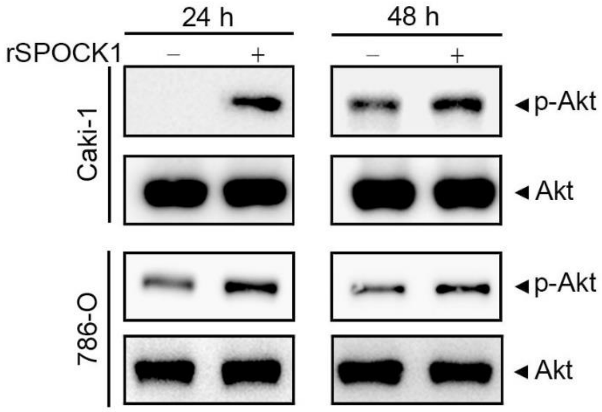

Figure S7. Effects of recombinant (r)SPOCK1 on Akt activation in clear cell renal cell carcinoma (ccRCC) cells. Treatment of Caki-1 and 786-O cells with or without the rSPOCK1 protein (1 ng/ml) for 24 and 48 h, and phosphorylation of Akt (p-Akt) was detected by Western blotting. Quantitative results of p-Akt were adjusted to total Akt protein levels.

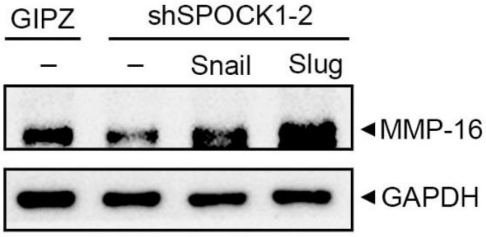

Figure S8. Effects of Snail family overexpression on matrix metalloproteinase (MMP)-16 expression in SPOCK1-knockdown clear cell renal cell carcinoma (ccRCC) cells. Snail, Slug, shSPOCK1, and their respective control vectors were respectively overexpressed in Caki-1 cells as indicated. Expression levels of MMP-16 were detected by a Western blot analysis. Quantitative MMP-16 levels were adjusted to GAPDH levels.

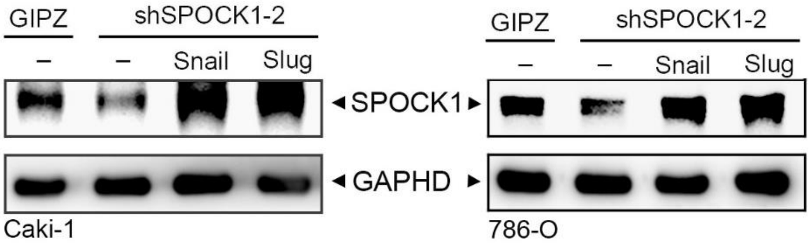

Figure S9. Effects of Snail family overexpression on SPOCK1 expression in SPOCK1-knockdown clear cell renal cell carcinoma (ccRCC) cells. Snail, Slug, shSPOCK1, and their respective control vectors were respectively overexpressed in Caki-1 and 786-O cells as indicated. Expression levels of SPOCK1 were detected by a Western blot analysis. Quantitative SPOCK1 levels were adjusted to GAPDH levels.
